# Green Preparation of Ag-Au Bimetallic Nanoparticles Supported on Graphene with Alginate for Non-Enzymatic Hydrogen Peroxide Detection

**DOI:** 10.3390/nano8070507

**Published:** 2018-07-08

**Authors:** Li Zhao, Yesheng Wang, Xihui Zhao, Yujia Deng, Qun Li, Yanzhi Xia

**Affiliations:** 1 School of Chemistry and Chemical Engineering, Qingdao University, Qingdao 266071, China; 2017020829@qdu.edu.cn (L.Z.); 2016020405@qdu.edu.cn (Y.W.); dengyujia@qdu.edu.cn (Y.D.); 2 State Key Laboratory Breeding Base of New Fiber Materials and Modern Textile, School of Materials Science and Engineering, Qingdao University, Qingdao 266071, China; xiayzh@qdu.edu.cn; 3 Collaborative Innovation Center for Marine Biomass Fibers, Materials and Textiles of Shandong Province, Institute of Marine Bionbased Materials, Qingdao University, Qingdao 266071, China

**Keywords:** Alginate, Ag-AuNPs/RGO, Non-enzymatic sensor, Hydrogen peroxide

## Abstract

In this work, a facile, environmentally friendly method was demonstrated for the synthesis of Ag-Au bimetallic nanoparticles (Ag-AuNPs) supported on reduced graphene oxide (RGO) with alginate as reductant and stabilizer. The prepared Ag-AuNPs/RGO was characterized by scanning electron microscope (SEM), transmission electron microscopy (TEM), X-ray diffraction (XRD), and X-ray photoelectron spectroscopy (XPS). The results indicated that uniform, spherical Ag-AuNPs was evenly dispersed on graphene surface and the average particle size is about 15 nm. Further, a non-enzymatic sensor was subsequently constructed through the modified electrode with the synthesized Ag-AuNPs/RGO. The sensor showed excellent performance toward H_2_O_2_ with a sensitivity of 112.05 μA·cm^−2^·mM^−1^, a linear range of 0.1–10 mM, and a low detection limit of 0.57 μM (S/N = 3). Additionally, the sensor displayed high sensitivity, selectivity, and stability for the detection of H_2_O_2_. The results demonstrated that Ag-AuNPs/RGO has potential applications as sensing material for quantitative determination of H_2_O_2_.

## 1. Introduction

As a green chemical product, hydrogen peroxide (H_2_O_2_) has a wide range of applications in environmental [1], clinical [2], pharmaceutical [3], food industry [4], textiles industry [5], and other fields. At the same time, H_2_O_2_ is also a byproduct of various enzyme catalyzed reactions [6]. Therefore, it is important to perform rapid, simple, and accurate detection of H_2_O_2_. Currently, many methods are used for the detection of H_2_O_2_, such as fluorometry [7], spectrophotometry [8], titrimetry [9], chemiluminescence [10], and electrochemical detection [11]. Among them, the electrochemical detection method has attracted wide attention due to its advantages of low cost, fast response, good stability, high sensitivity, and strong selectivity [12]. Additionally, electrochemical detection methods mainly include two categories: enzymatic detection and non-enzymatic detection [13]. The most prominent feature of the enzyme sensor is good selectivity, but there are also expensive enzymes, active environment interference, poor stability, short service life, and other issues. Therefore, the research on non-enzymatic H_2_O_2_ sensors is significant.

Nanomaterials exhibit good catalytic performance due to its unique structure and morphology. In recent years, the use of nanomaterials for sensor research has attracted widespread interest [14]. A variety of metal nanoparticles, such as PtNPs [15], AuNPs [16], AgNPs [17], and SnNPs [18] have been reported to modify non-enzymatic sensors. Among them, many studies focus on Ag and Au nanoparticles due to their high catalytic activities, excellent bio-compatibility, ease of synthesis, and strong absorption property in the visible region [19]. Additionally, the bimetallic nanoparticles have better catalytic activity than the corresponding single metal nanoparticles because of their special optical, catalytic and electrical properties [2,14,20]. Li et al. synthesized Au@AgNPs via seed-mediated growth procedure for sensitive detection of H_2_O_2_. Although metal nanoparticles has excellent electrocatalytic activities, which can be used for determine H_2_O_2_ with remarkable voltammetric responses, the easy aggregation tend to significantly decrease their catalytic activity and selectivity [10,21]. In order to overcome the aggregation of nanoparticles, various supporting materials, like resins, fibers, carbon nanotube, graphene etc., have been used to disperse nanoparticles [22]. It is noteworthy that graphene, as a support material for nanoparticles, has attracted wide attention because of its good structural stability, large specific surface area, fast electron conduction rate and large mechanical strength [23]. Additionally, graphene can be used as a matrix for various metal nanoparticles due to the honeycomb-like structural characteristics, which makes it easier for carbon atoms to participate in catalytic reactions. A large number of studies have shown that graphene/metal nanoparticles composites not only have excellent physical and chemical properties of two-dimensional graphene structure, but also have the high catalytic activity and high electron transfer rate of metal nanomaterials [24]. Therefore, electrochemical sensors based on graphene/metal nanoparticles composites will have excellent sensory characteristics and important application values [25].

Sodium alginate, extracted from brown algae, is a kind of natural polysaccharide carbohydrate, and is an anionic copolymer of β-d-mannuronic acid (M) and α-l-guluronic acid (G) units. Sodium alginate contains a large number of carboxyl groups, hydroxyl, as well as carbonyl group formed by glycosidic bond cleavage, making it both as a stabilizer and a reducing agent in the preparation of metal nanoparticles [26].

At the same time, sodium alginate is also a renewable resource with certain biocompatibility, non-toxic and low cost [27,28]. Although many literatures have reported the metal nanoparticles doped on graphene, toxic reducers and stabilizers have been used [19,29]. Additionally, so far as we know, no attention has been paid to the preparation of Au-AgNPs loaded on reduced graphene oxide (RGO) with alginate as stabilizer and reducing agent. 

In this paper, sodium alginate is used as a stabilizer and a reducing agent to reduce metal ions into metal nanoparticles and loaded them on RGO. A non-enzymatic sensor was fabricated by modifying glassy carbon electrode with the prepared Ag-AuNPs/RGO, which was applied for electrochemical detection of H_2_O_2_. The linearity, sensitivity, selectivity, and stability of the sensor were investigated via cyclic voltammetry and chronoamperometry.

## 2. Experimental

### 2.1. Reagents

Reduced graphene oxide (RGO) was supplied by Huagao Graphene Co. Ltd (Qingdao, China) Chloroauric acid (HAuCl_4_), sodium alginate, 30% hydrogen peroxide (H_2_O_2_) were purchased from Sinopharm Group Chemical Reagent Co. Ltd (Beijing, China). Silver nitrate (AgNO_3_) was obtained from Shanghai Fine Chemical Research Institute (Shanghai, China). All other reagents were of analytical grade and used without further purification. All solutions were prepared with Ultra-pure water.

### 2.2. Preparation of Ag-Au Nanoparticles (NPs)/ Reduced Graphene Oxide (RGO)

In a typical experiment, Ag-AuNPs/RGO was prepared by a facile solvothermal process. First, 1.5 mg of graphene was added to 5 mL membrane-filtered sodium alginate solution (0.5%) and sonicated for 1 h. Then, 40 μL of a 24 mmol/L AgNO_3_ was added to the mixture, sonicated for 30 min, the mixed solution was heated at 90 °C for 3 h. After the reaction is complete, 160 μL of a 24 mmol/L HAuCl_4_ precursor was added to the mixture, sonicated for 30 min, and incubated at 90 °C for 1 h. Finally, the resulting mixture was centrifuged at 15,000 r/min and washed with Ultra-pure water for several times. The final product was denoted as Ag-AuNPs/RGO. For comparison, the AuNPs/RGO was further prepared with 200 μL HAuCl_4_ (24 mmol/L) precursor heated at 90 °C for 1 h, and the rest of the processes were the same.

### 2.3. Characterization and Measurement

The scanning electron microscope (SEM) measurements were performed using a JSM-7800F (JEOL, Tokyo, Japan) system. Transmission electron microscopy (TEM) image analysis was performed on a JSM-2100Plus (JEOL, Tokyo, Japan) microscope and energy dispersive X-ray spectroscopy (EDS) was obtained from the same apparatus. X-ray diffraction (XRD) patterns were recorded on a powder X-ray diffractometer (D/MAX-RB, Tokyo, Japan) measured with CuKα radiation (*λ* = 0.15418 nm) in the 2θ range of 10° to 80°. X-ray photoelectron spectroscopy (XPS) analysis was carried out on an ESCALAB 250 electronic spectrometer (ThermoFisher, Waltham, MA, USA).

### 2.4. Preparation of the Modified Electrode

The Ag-AuNPs/RGO modified glassy carbon electrode (GCE) was prepared as follows. Firstly, the glassy carbon electrode was thoroughly polished with alumina powder and then cleaned with water and ethanol in an ultrasonic bath [30]. Secondly, 20 μL of the prepared dispersion was dropped onto a pre-treated GCE and dried at room temperature. All of the samples were made in the same process to keep the same loading. Electrochemical measurements were carried out at room temperature. The traditional three electrode system was controlled by CHI 760E electrochemical workstation (CH Instrument Co., Ltd., Shanghai, China). The Ag-AuNPs/RGO modified electrode, the saturated calomel electrode (SCE) and the platinum wire were used as the working electrode, reference electrode and counter electrode, respectively [31,32]. With the same process, the AuNPs/RGO modified GCE was also prepared. Since the loading of all the samples were the same, the comparisons of the currents without normalization could lead to the same results as those of the normalized ones.

## 3. Results and Discussion

### 3.1. Characterization of Ag-AuNPs/RGO

**Scheme 1:**
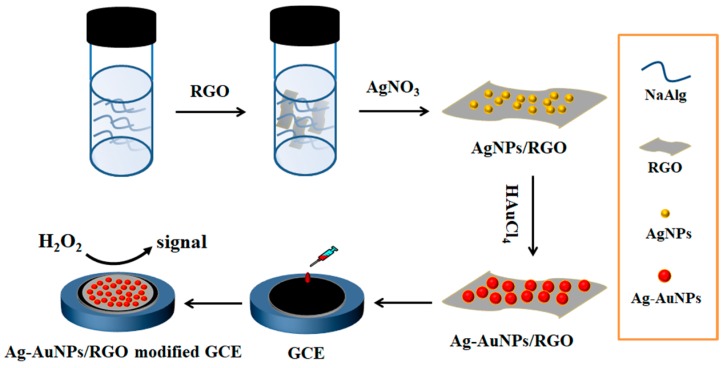
Schematic illustration of synthesis of Ag-AuNPs/reduced graphene oxide (RGO) and its application in the electrochemical detection of H_2_O_2_.

The synthetic strategy of Ag-AuNPs/RGO is presented in Scheme 1. Firstly, AgNO_3_, alginate and RGO were uniformly mixed. AgNPs/RGO nanocomposite was synthesized via the reduction of Ag^+^ by alginate to form Ag nanoparticles and loaded on RGO surface. Here, sodium alginate plays important roles, both as reducing and stabilizing agent. Additionally, hydroxyl (–OH) and carboxyl (–COO–) groups of alginate act as inter linkers contributing to the deposition of silver nanoparticles on the graphene surface. Then, AgNPs would serve as nucleation sites and HAuCl_4_ was directly reduced around AgNPs on graphene. It is worth noting that, in the whole process, Ag-AuNPs/RGO was prepared without the aid of surfactant, so that the metal nanoparticles catalyst was completely exposed during the catalytic process, which is conducive to the direct contact of the catalyst [33,34]. Scheme 1 illustrated the synthesis of Ag-AuNPs/RGO and its application in the electrochemical detection of H_2_O_2_.

RGO, AgNPs/RGO, AuNPs/RGO, and Ag-AuNPs/RGO were measured by ultraviolet-visible (UV-Vis) spectroscopy (T9 spectrophotemeter, Beijing, China) and the result was shown in Figure S1. The UV-Vis absorption spectrum of RGO was showed in Figure S1a without obvious absorption peaks. The characteristic peaks of AgNPs/RGO, AuNPs/RGO appeared at 438 and 540 nm, respectively (Figure S1b,c). However, there is no such absorption peaks in (Figure S1d). Ag-AuNPs/RGO showed a new absorption peak at 524 nm, the only one Plasmon band confirms the formation Ag-Au bimetallic nanopaticles on the RGO surface.

**Figure 1:**
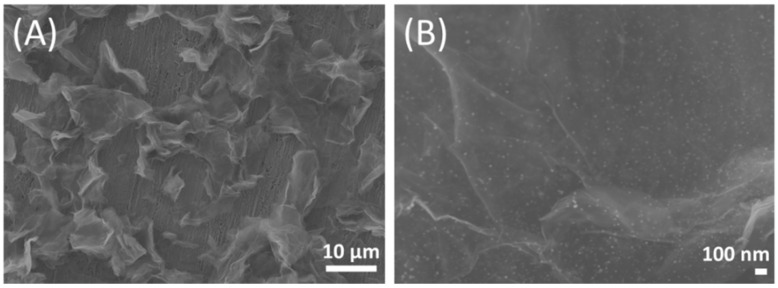
Scanning electron microscope (SEM) images of Ag-AuNPs/RGO magnified 1500 times (**A**); and 33,000 times (**B**).

In addition, metal nanoparticles are easy to aggregate in solution, which will damage their catalytic ability. However, as intuitively shown in the SEM images of Ag-AuNPs/RGO (Figure 1), the metal nanoparticles were uniformly dispersed on RGO surface without obvious agglomeration, indicating that Ag-AuNPs was loaded on RGO to avoid aggregation. Moreover, RGO are thin lamellar structure with folded wrinkles, and the uniformity of the graphene layer was maintained even after doping with Ag-AuNPs, indicating that Ag-AuNPs were embedded in the RGO framework to prevent stacking. The synergistic effect of RGO carrier and Ag-AuNPs leads to the formation of the three-dimensional conductive network of Ag-AuNPs/RGO, which not only could speed up the reaction rate, but also widens the space of charge transfer in the catalytic process [33].

**Figure 2:**
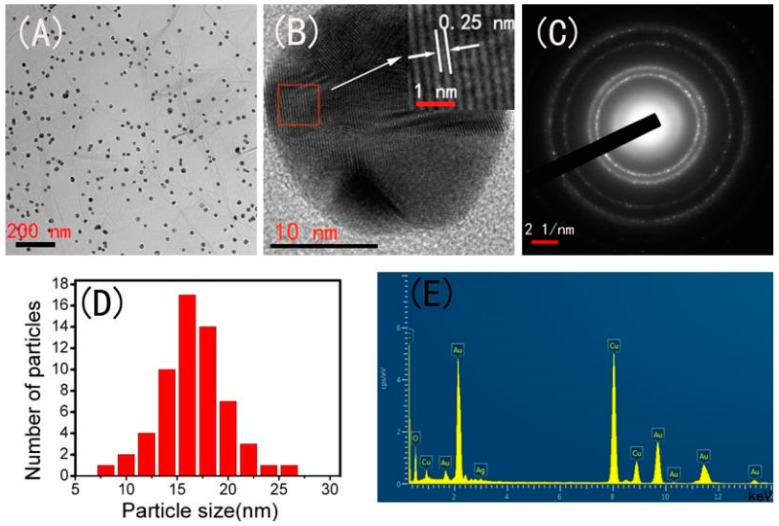
(**A**) Transmission electron microscopy (TEM) image; (**B**) HR-TEM image; (**C**) selected area electron diffraction (SAED) pattern; (**D**) the size distribution histogram; and (**E**) energy dispersive X-ray spectroscopy (EDS) spectrum of Ag-AuNPs/RGO.

The shape and size of the Ag-AuNPs on RGO sheet surfaces were determined by TEM analysis. As shown in Figure 2A, it can be seen that spherical Ag-Au bimetallic nanoparticles are uniformly supported on RGO, and almost no aggregation, mainly due to the synergistic effect of sodium alginate and graphene [29]. The high resolution transmission electron microscope (HRTEM) image of Ag-AuNPs was shown in Figure 2B, clear lattice fringes could be observed with a interplanar distance of 0.25 nm, which is attributed to (111) planes of Ag-AuNPs [35]. The selected area electron diffraction (SAED) pattern of Ag-AuNPs/RGO with bright circular rings in Figure 2C confirmed that the synthesized Ag-AuNPs were crystalline in nature. The size distribution histogram was given in Figure 2D with the average particle size 15 nm. The initial formed AgNPs/RGO was also investigated and the distribution of AgNPs on RGO was shown in Figure S2. The average size is about 10 nm, which is smaller than Ag-AuNPs. In addition, the chemical composition of the Ag-AuNPs/RGO was further examined by energy-dispersive X-ray spectrum (EDS) analysis (Figure 2E), confirming the presence of Ag and Au elements on RGO surface. These results clearly suggest that uniformly dispersed Ag-AuNPs with small particle size were anchored on the surface of RGO and RGO are excellent carrying materials to load Ag-AuNPs. Furthermore, EDS quantitative elemental analysis was supplied in Table S1. The atomic percent of Ag and Au in Ag-AuNPs/RGO is 0.37% and 4.02%, respectively. The ration of Ag-Au is lower than the initial ratio of the two precursors. The possible reason is that partial Ag is oxidized to Ag^+^ by Au ions due to the standard reduction potential of Ag^+^/Ag (0.80 V vs SHE) is lower than that of AuCl_4_^−^/Au (0.99 V vs SHE) [22]. 

For comparison, AuNPs/RGO was also synthesized and observed with TEM (Figure S3A). Compared with Ag-Au/RGO, AuNPs have larger particle sizes and have a variety of morphologies. We selected a special form of AuNPs for HRTEM analysis (Figure S3B). The obtained AuNPs showed clear fringes with a pitch of 0.24 nm, which matches the d-spacing of the (111) face of the face centered cubic (fcc) Au. This result also indicated the crystalline structure of AuNPs. Furthermore, the corresponding fast Fourier transform (FFT) pattern (Figure S3C) confirmed the single crystal nature. The size distribution histogram of AuNPs was given in Figure S3D, the average particle size was 28 nm, larger than Ag-AuNPs. By comparison, we can see that Ag-AuNPs are smaller and more uniform than those of AuNPs, which has great influence on the catalytic activity to H_2_O_2_ detection [36]. 

**Figure 3:**
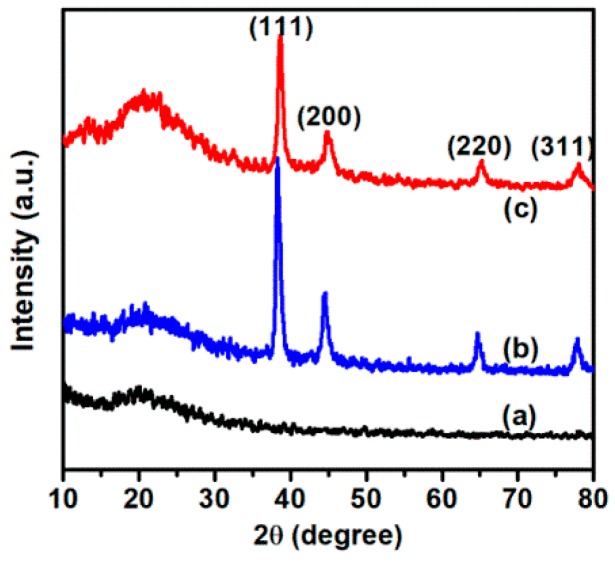
X-ray diffraction (XRD) patterns of RGO (**a**); AuNPs/RGO (**b**); and Ag-AuNPs/RGO (**c**).

The XRD patterns of RGO, AuNPs/RGO, and Ag-AuNPs/RGO are shown in Figure 3, and data were collected in the angular range 10° ≤ 2θ ≤ 80° [37]. In the XRD pattern of RGO, the diffraction peaks observed at 20° was assigned to the (002) facet of crystalline RGO. In Ag-AuNPs/RGO diagram, four strong diffraction peaks at 38.4°, 44.3°, 64.5°, and 77.5°, representing the Bragg reflection from (111), (200), (220), and (311), respectively, which were assigned to the crystal plane of bimetallic Ag-Au [32]. AuNPs/RGO showed four peaks at 38.1°, 44.1°, 64.3°, and 77.2°, which were attributed to the diffraction of (111), (200), (220), and (311) planes, respectively, of the face centered cubic (FCC) structure of Au (JCPDS number: 04-0784) [38]. Ag-AuNPs/RGO and AuNPs/RGO showed similar XRD pattern due to AgNPs and AuNPs have same diffraction planes at similar Bragg’s angle. 

**Figure 4:**
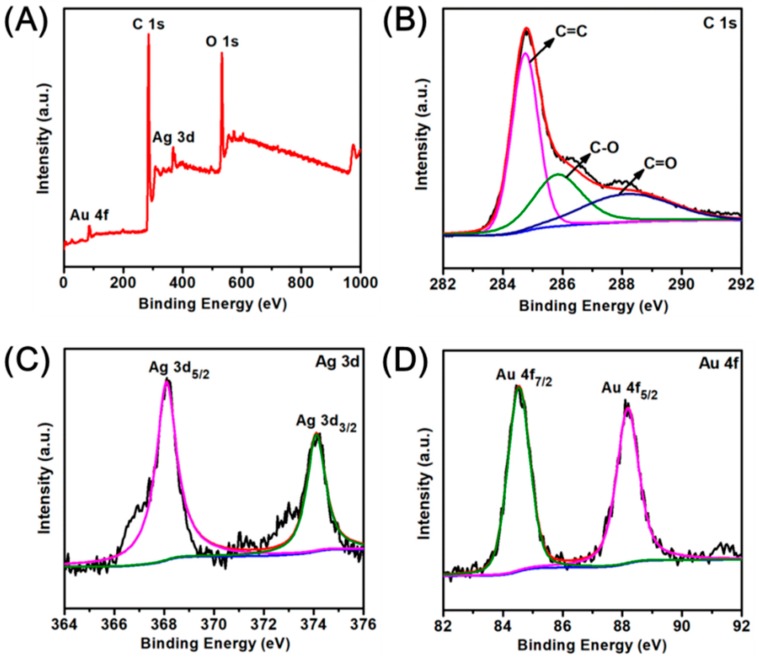
(**A**) X-ray photoelectron spectroscopy (XPS) full scan spectrum of Ag-AuNPs/RGO; (**B**–**D**) high resolution XPS spectrum for C 1s; Ag 3d; and Au 4f, repectively.

XPS was further used to investigate the chemical composition and the chemical states of different elements of Ag-AuNPs/RGO [39]. Figure 4A showed the survey scans spectrum, confirming the presence of elements C, O, Ag, and Au in the as-prepared Ag-AuNPs/RGO, which is consistent with the EDS report. Two peaks observed at binding energy of 284.8 and 532.8 eV are attributed to C 1s and O 1s bands, respectively. Additionally, The C 1s high-resolution scan spectrum (Figure 4B) could be deconvoluted into three peaks at 284.6, 286.4 and 288.8 eV, which are associated with C=C, C–O, and C=O, respectively. High resolution XPS of Ag 3d (Figure 4C) showed 6 eV splitting double peaks at 368.1 and 374.2 eV, corresponding to Ag 3d_5/2_ and Ag 3d_3/2_ energy levels of the Ag atom, respectively, these binding energies indicated the metallic nature of silver [13]. At the same time, high-resolution XPS of Au 4f in Figure 4D presented two peaks at binding energies of about 84.3 and 87.8 eV for Au 4f_7/2_ and 4f_5/2_, which match well to the standard binding energy of the Au atom (84.0 and 87.6 eV) [40]. XPS results further indicated Ag and Au ions were reduced to Ag-AuNPs. In the supplementary information, we also give the XPS plots of AuNPs/RGO (Figure S4). The full scan XPS of AuNPs/RGO consist of C, O, and Au special peaks (Figure S4A). The XPS scan of C 1s (Figure S4B) could also be deconvoluted into three peaks at 284.6 (C=C), 286.4 (C–O), and 288.8 eV(C=O), respectively. The Au 4f spectrum revealed two peaks at about 84.3 and 87.9 eV for Au 4f_7/2_ and 4f_5/2_ (Figure S4C), indicating the metallic nature of gold.

### 3.2. Electrochemical Behaviors of the Ag-AuNPs/RGO

**Figure 5:**
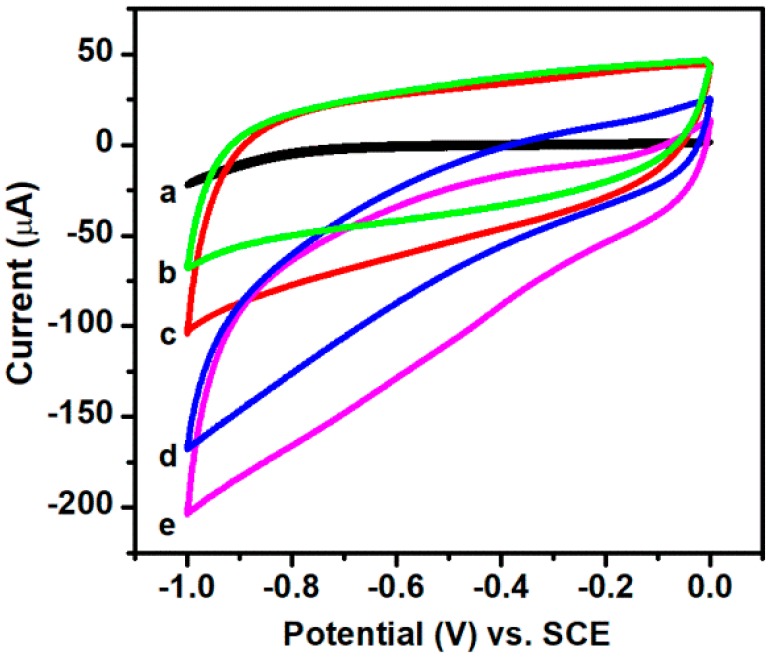
Cyclic voltammetry (CV) curves of bare GCE obtained in the presence of 3 mM H_2_O_2_ (**a**); CV curves of AuNPs/RGO modified electrode obtained in the absence (**b**) and presence of 3 mM H_2_O_2_ (**d**); and Ag-AuNPs/RGO modified electrode in the absence (**c**) and presence of 3 mM H_2_O_2_ (**e**). Scan rate: 50 mV·s^−1^.

In order to study the sensing properties of nanocomposites prepared, a non-enzymatic hydrogen peroxide sensor was constructed by depositing samples directly on the bare GCE surface. The electrochemical properties of AuNPs/RGO modified electrode, Ag-AuNPs/RGO modified electrode were investigated by cyclic voltammetry (CV) in Ar-saturated 0.1 mM phosphate buffered solution (PBS) (pH = 7.2) at a scan rate of 50 mV·s^−1^. Figure 5 shows the electrochemical response of bare GCE, AuNPs/RGO modified electrode and Ag-AuNPs/RGO modified electrode. As can be seen from the results, the reduction current of bare GCE (curve a) is almost zero even though adding 3 mM hydrogen peroxide. However, although no reduction peak appeared, the modified electrode has a significant current response due to the good conductivity of the metal nanoparticles. When H_2_O_2_ is not present, the reduction currents of AuNPs/RGO modified electrode (curve b) and Ag-AuNPs/RGO modified electrode (curve d) are small, but after H_2_O_2_ addition, the reduction current increases significantly, confirming the catalytic activity of metal nanoparticles for electrochemical reduction of H_2_O_2_. Notably, Ag-AuNPs/RGO modified electrode exhibits stronger current response (curve e) in comparison to AuNPs/RGO modified electrode (curve c), suggesting bimetallic catalyst with larger active area may provide more catalytic active sites than single metal catalysts [41,42].

**Figure 6:**
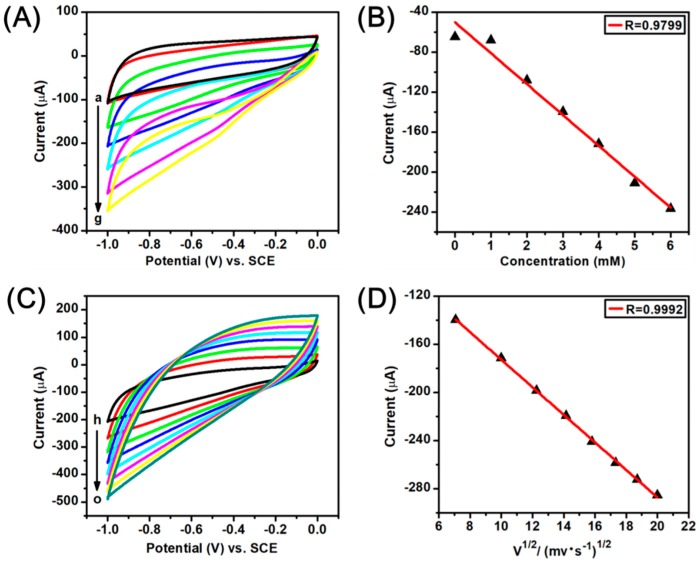
(**A**) CV curves of Ag-AuNPs/RGO modified GCE obtained in Ar-saturated 0.1 M PBS (pH 7.2) with different concentration H_2_O_2_ (a–g: 0, 1, 2, 3, 4, 5 and 6 mM), scan rate: 50 mV·s^−1^; (**B**) Linear fitting curve of the reduction peak current (−0.65 V) vs. the H_2_O_2_ concentration; (**C**) CV curves of Ag-AuNPs/RGO modified GCE in Ar-saturated 0.1M PBS (pH 7.2) containing 3mM H_2_O_2_ at different scan rates (h–o: 50, 100, 150, 200, 250, 300, 350, and 400 mV·s^−1^); (**D**) the corresponding calibration plot of the reduction peak current (−0.65 V) vs. the square root of scan rate.

More CV experiments were conducted to study the H_2_O_2_ catalysis on the Ag-AuNPs/RGO modified electrode surface. Figure 6A displays the CV curves of Ag-AuNPs/RGO modified GCE at different H_2_O_2_ concentration in 0.1 mM PBS (pH = 7.2), and scan rate is 50 mV·s^−1^. It can be seen that the reduction current gradually increases with increasing concentration of H_2_O_2_, which indicates that the prepared Ag-AuNPs/RGO have a good catalytic response toward the changing of the concentration of H_2_O_2_. A good linear relationship between peak current and H_2_O_2_ concentration from 1.0 to 6.0 mM is shown in Figure 6B, the linear correlation coefficient is 0.9799 (*R* = 0.9799). In order to study possible kinetic mechanisms, we further tested the CV curves of Ag-AuNPs/RGO modified GCE at different scan rate. As shown in Figure 6C, with the increase of scanning rate from 50 to 400 mV·s^−1^, the electrocatalytic current gradually increases. Moreover, there is a good linear relationship between the response current and the square root of the scan rate (showed in Figure 6D), and the linear correlation coefficient *R* = 0.9992, suggesting that the reduction of H_2_O_2_ on the Ag-AuNPs/RGO modified GCE surface is diffusion control process. In addition, the CV experiments of the AuNPs/RGO modified GCE were also investigated, and the results were presented in Figure S5. By comparison, it is found that the bimetallic sensor reveals better performance than the single gold sensor. This may be due to the synergy of gold and silver.

**Figure 7:**
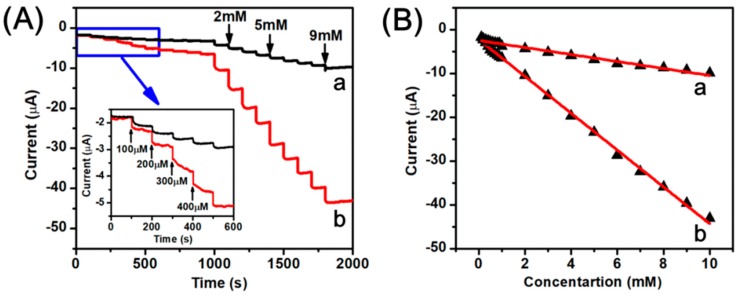
(**A**) Amperometric response of the AuNPs/RGO (a) and the Ag-AuNPs/RGO (b) sensors upon successive addition of H_2_O_2_ into 0.1 M PBS, pH 7.2 under a stirring condition, applied potential −0.2 V. The inset was the amplification of curve with the lower concentration region; (**B**) The linear relationship between different H_2_O_2_ concentration and reduction current.

Figure 7A shows the amperometric current-time curves for AuNPs/RGO modified GCE (curve a) and Ag-AuNPs/RGO modified GCE (curve b). The experiment was carried out in the Ar-saturated PBS (pH 7.2) solution with successive injections of H_2_O_2_. According to the previous reports, the higher potential could reduce noise and improve the sensing performance, so the constant potential of −0.2 V was applied [42]. It can be observed that the current response to successive injections of H_2_O_2_ is significant. Moreover, the Ag-AuNPs-RGO modified GCE has a stronger current response than the AuNPs-RGO modified GCE, which further indicates that the Ag-AuNPs/RGO modified electrode exhibits better electrochemical performance than the AuNPs/RGO modified electrode. Additionally, after each addition of H_2_O_2_, Ag-AuNPs/RGO modified GCE transiently responds to hydrogen peroxide, and the reduction current drastically increases to a stable value. Figure 7B shows a linear relation between the steady state reduction current and the concentration of H_2_O_2_ with a linear range of 0.1–10 mM. The correlation coefficient for AuNPs/RGO (curve a), Ag-AuNPs/RGO (curve b) are 0.9800 and 0.9981, respectively. The estimated sensitivity for H_2_O_2_ sensor was 112.05 μA·cm^−2^·mM^−1^. Additionally, according to the 3σ rule, the detection limit was calculated to be 0.57 μM estimated at a signal-to-noise ratio of 3 (*S*/*N* = 3), which was the value of 3 σ/*S* (σ is the standard deviation of the background signal, and *S* is the slope of H_2_O_2_ concentration and reduction current shown in the Figure 7B(b) [12,43,44,45,46]. The performances of modified electrodes were also compared with various sensors based on Ag and Au nanomaterials, and the results were presented in Table 1. The performance of our sensor herein is comparable and even better compared to other previous reported.

### 3.3. Stability and Interference Study on the Ag-AuNPs/RGO Modified Electrode

**Figure 8:**
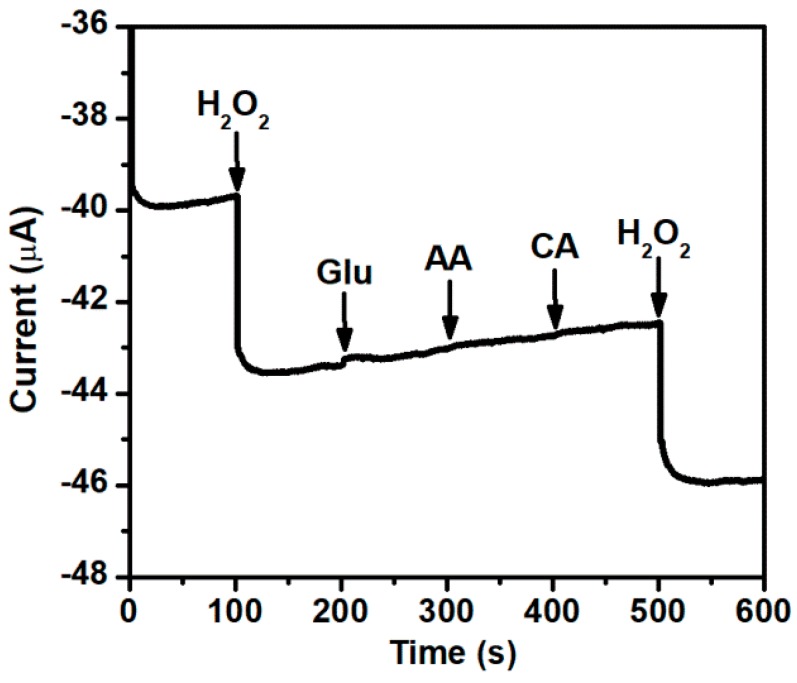
Amperometric response of the Ag-AuNPs/RGO modified GCE for the successive addition of H_2_O_2_, glucose, AA and CA (1 mM, respectively) in Ar-saturated 0.1 M PBS (pH 7.2) at −0.2 V.

In order to investigate the stability of Ag-AuNPs/RGO modified electrode, 50 continuous cycles were performed at a scan rate of 50 mV·s^−1^ in PBS (pH 7.2) containing 3 mM of H_2_O_2_. The result shows only 2.0% loss from the initial determination value (Figure S6). This result demonstrates that the Ag-AuNPs/RGO modified electrode has a good stability and repeatability. These excellent properties may be mainly attributed to the excellent stability of Ag-AuNPs on the surface of RGO. Furthermore, chronoamperometry was used to investigate the selectivity and anti-interference ability of Ag-AuNPs/RGO modified electrode as a hydrogen peroxide sensor. As shown in Figure 8, Ag-AuNPs/RGO electrode showed no significant current response to Glu (glucose), AA (ascorbic acid), and CA (citrate acid), but had a significant current response to H_2_O_2_, confirming that the modified GCE with Ag-AuNPs/RGO has good selectivity to H_2_O_2_ and good anti-interference ability to electroactive substances. Consequently, all these excellent properties provide potential applications for Ag-AuNPs/RGO as a non-enzymatic sensing material. 

## 4. Conclusions

In this study, we used sodium alginate as a reducing agent and stabilizer, and graphene as a carrier to prepare uniform Ag-AuNPs with good stability. From the electron microscopy image, it can be seen that the Ag-AuNPs are evenly supported on the surface of RGO without apparent aggregation. The nanoparticles are uniformly distributed, with particle sizes ranging from 8 to 30 nm and spherical in shape. A non-enzymatic H_2_O_2_ sensor was successfully constructed by dropping Ag-AuNPs/RGO on GCE. Compared with other studies, the sensor exhibits good electrocatalytic response for H_2_O_2_, and also has a wider linear range, lower detection limit and high sensitivity. In addition, the sensor also has special selectivity for hydrogen peroxide.

## Figures and Tables

**Table 1:** Comparison of analytical performance of Ag-AuNPs-RGO modified electrode with previously reported similar modified electrodes for the detection of H_2_O_2_.

Modified Eletrode	Applied Potential (V)	Linear Range (mM)	Sensitivity (µA·mM^−1^·cm^−2^)	Detection Limit (μM)	Ref.
Ag Nanowires	−0.2	0.1–3.1	26.6	29.2	[46]
Ag NPs-NFs ^a^/GCE ^b^	−0.3	0.1–80	—	62	[47]
ERGO ^c^-Ag/GCE	−0.3	0.1–100	—	1.6	[48]
Ag Microspheres	−0.5	0.25–2.0	—	1.2	[49]
Porous Ag	−0.2	0.5–4.5	31.8	29.8	[12]
AuNPs-GO/Pt	0.4	0.05–4.6	31.47	25	[16]
Ag-rGO-CNT/ITO ^d^	−0.2	0.05–1.4	—	1.32	[42]
Ag-AuNPs-RGO	−0.2	0.1–10	112.05	0.57	this work

^a^ NFs: nanofibers. ^b^ GCE: glassy carbon electrode. ^c^ ERGO: electrochemically reduced graphene oxide. ^d^ CNT/ITO: carbon nanotube/indium tin oxide.

## Supplementary Materials

The following are available online at http://www.mdpi.com/2079-4991/8/7/507/s1, Figure S1. Ultraviolet-visible (UV-Vis) absorption spectra of (**a**) RGO; (**b**) AgNPs/RGO; (**c**) AuNPs/RGO and (**d**) Ag-AuNPs/RGO. Table S1. Energy dispersive X-ray spectroscopy (EDS) quantitative elemental analysis of Ag-AuNPs/RGO. Figure S2. Transmission electron microscopy (TEM) image and the size distribution histogram of AgNPs/RGO. Figure S3. (**A**) TEM image; (**B**) high resolution transmission electron microscope (HR-TEM )image; (**C**) fast fourier transform (FFT) pattern; and (**D**) the size distribution histogram of AuNPs/RGO. Figure S4. X-ray photoelectron spectroscopy (XPS) of AuNPs/RGO: (**A**) survey scan; (**B**) high resolution scans for C 1s; and (**C**) Au 4f. Figure S5. (**A**) Cyclic voltammetry (CV) curves of AuNPs/RGO modified electrode obtained in Ar-saturated 0.1 M PBS (pH 7.2) in the absence and presence of H_2_O_2_ with different concentration (from a to g: 0, 1, 2, 3, 4, 5 and 6 mM) at a 50 mV·s^−1^ scan rate; (**B**) Linear fitting program of the reduction peak current (-0.65 V) versus the H_2_O_2_ concentration; (**C**) CV curves of AuNPs/RGO modified electrode in Ar-saturated 0.1M PBS (pH 7.2) containing 3 mM H_2_O_2_ at different scan rates (from h to o: 50, 100, 150, 200, 250, 300, 350 and 400·mV·s^−1^); (**D**) Linear fitting program of the reduction peak current (−0.65 V) versus the square root of scan rate. Figure S6. Cyclic voltammetric stability of Ag-AuNPs/RGO modified GCE in Ar-saturated 0.1 M PBS (pH 7.2) containing 3mM of H_2_O_2_ at a scan rate of 50 mV·s^−1^.
