# Supplementary material for: Green Preparation of Ag-Au Bimetallic Nanoparticles Supported on Graphene with Alginate for Non-Enzymatic Hydrogen Peroxide Detection
Source: Nanomaterials (Basel). 2018 Jul 8;8(7):507. doi: 10.3390/nano8070507 (PMC6071074; doi:10.3390/nano8070507)
Supplement: Supplementary file 1 [file nanomaterials-08-00507-s001.docx]

**Supplementary Material**

**Green preparation of Ag-Au bimetallic nanoparticles supported on graphene with alginate for non-enzymatic hydrogen peroxide detection**

Li Zhao^1^, Yesheng Wang^1^, Xihui Zhao^1,3,*^, Yujia Deng^1^, Qun Li^1,*^, Yanzhi Xia^2,3^





**Figure S1.** Ultraviolet-visible (UV-Vis) absorption spectra of (**a**) RGO; (**b**) AgNPs/RGO; (**c**) AuNPs/RGO and (**d**) Ag-AuNPs/RGO.

**Table S1** Energy dispersive X-ray spectroscopy (EDS) quantitative elemental analysis of Ag-AuNPs/RGO.

| Element | Weight % | Atomic % |
| --- | --- | --- |
| C K | 24.76 | 65.45 |
| O K | 3.97 | 7.87 |
| Cu K | 44.97 | 22.29 |
| Ag K | 1.25 | 0.37 |
| Au L | 25.05 | 4.02 |
| Totals | 100.00 | 100.00 |


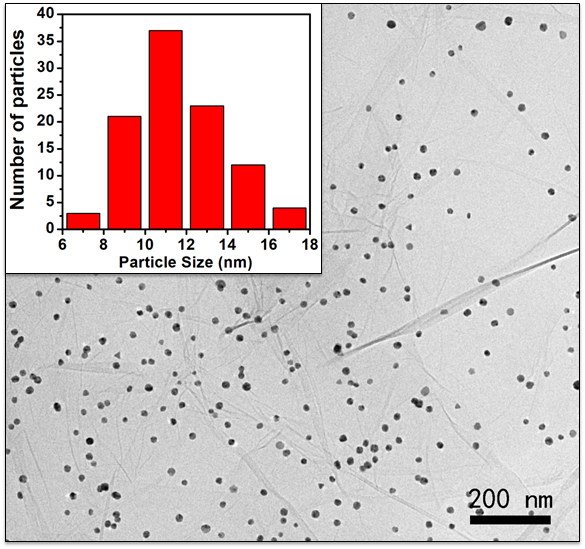


**Figure S2.** Transmission electron microscopy (TEM) image and the size distribution histogram of AgNPs/RGO.


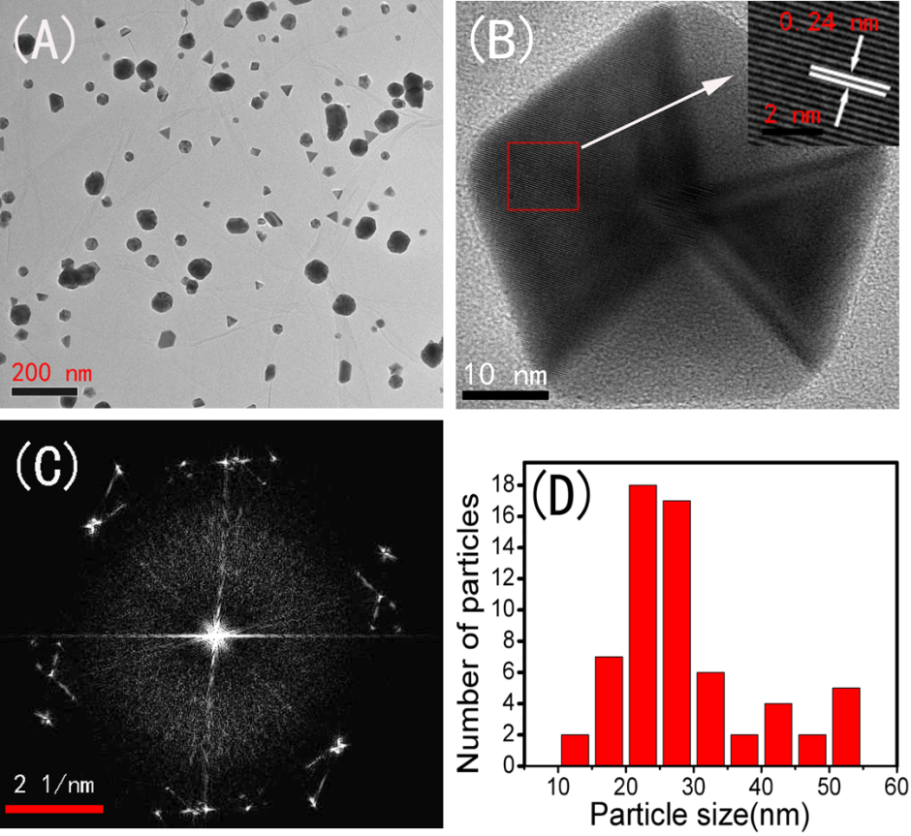


**Figure S3.** (**A**) TEM image; (**B**) high resolution transmission electron microscope (HR-TEM )image; (**C**) fast fourier transform (FFT) pattern; and (**D**) the size distribution histogram of AuNPs/RGO.

**
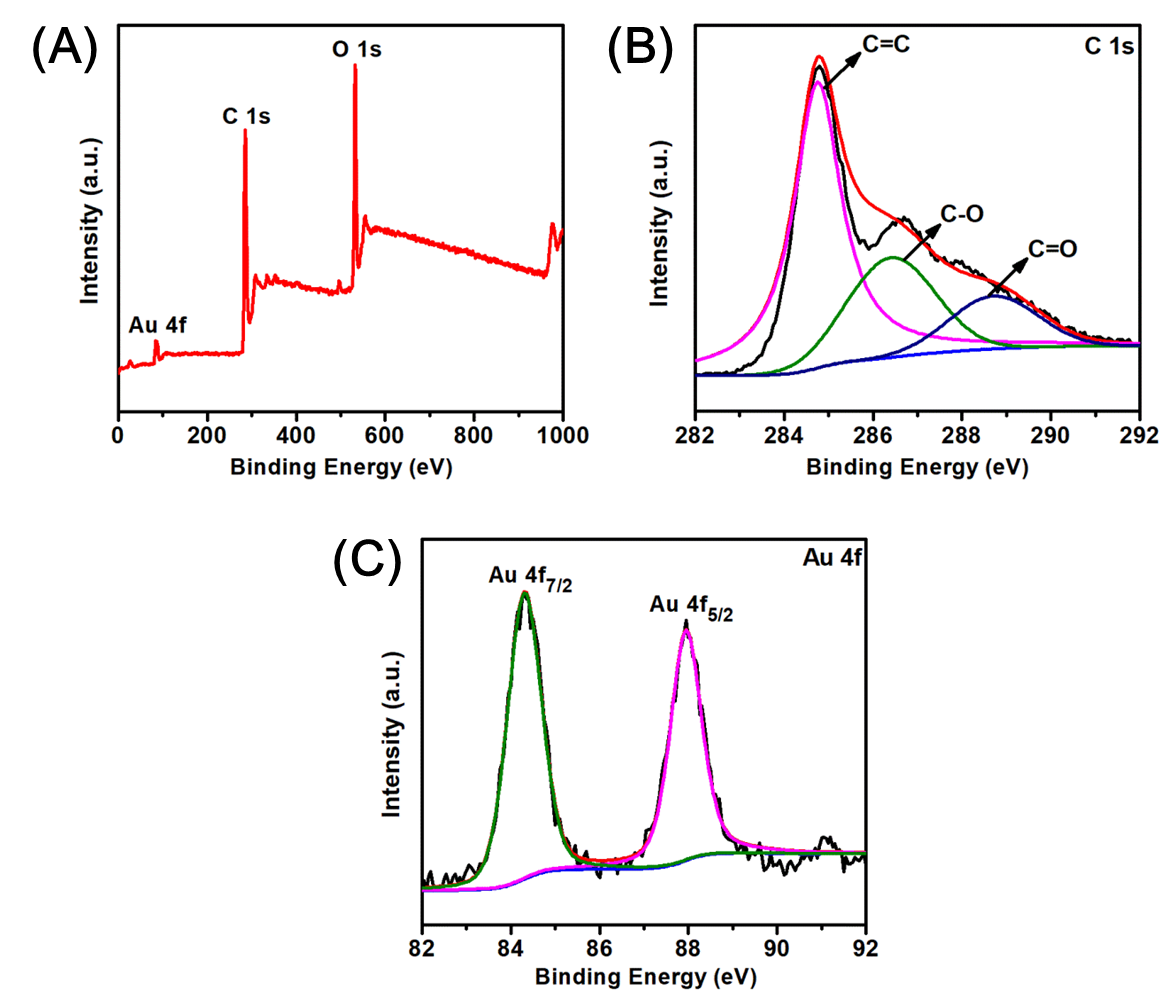
**

**Figure S4.** X-ray photoelectron spectroscopy (XPS) of AuNPs/RGO: (**A**) survey scan; (**B**) high resolution scans for C 1s; and (**C**) Au 4f.


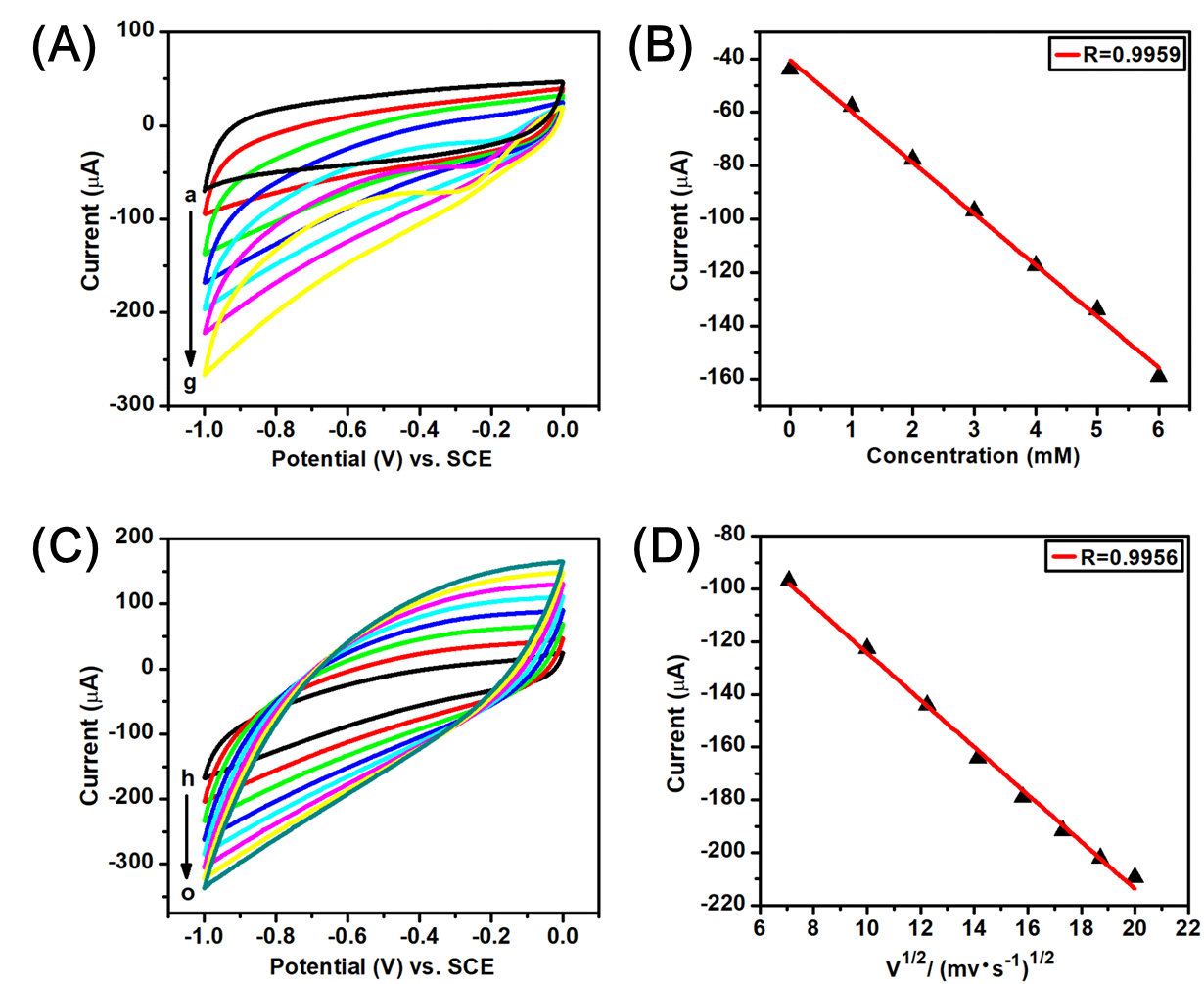


**Figure S5.** (**A**) Cyclic voltammetry (CV) curves of AuNPs/RGO modified electrode obtained in Ar-saturated 0.1 M PBS (pH 7.2) in the absence and presence of H_2_O_2_ with different concentration (from a to g: 0, 1, 2, 3, 4, 5 and 6 mM) at a 50 mV·s^−1^ scan rate; (**B**) Linear fitting program of the reduction peak current (-0.65 V) versus the H_2_O_2_ concentration; (**C**) CV curves of AuNPs/RGO modified electrode in Ar-saturated 0.1M PBS (pH 7.2) containing 3mM H_2_O_2_ at different scan rates (from h to o: 50, 100, 150, 200, 250, 300, 350 and 400·mV·s^−1^); (**D**) Linear fitting program of the reduction peak current (−0.65 V) versus the square root of scan rate.

**

**

**Figure S6** Cyclic voltammetric stability of Ag-AuNPs/RGO modified GCE in Ar-saturated 0.1 M PBS (pH 7.2) containing 3mM of H_2_O_2_ at a scan rate of 50 mV·s^−1^.
